# Sociodemographic gradients in breast and cervical cancer screening in Korea: the Korean National Cancer Screening Survey (KNCSS) 2005-2009

**DOI:** 10.1186/1471-2407-11-257

**Published:** 2011-06-17

**Authors:** Mi Jin Park, Eun-Cheol Park, Kui Son Choi, Jae Kwan Jun, Hoo-Yeon Lee

**Affiliations:** 1 National Cancer Control Institute, National Cancer Center, 111, Jungbalsan-ro, Ilsandong-gu, Goyang-si, Gyeonggi-do, 410-769, Korea; 2 Department of Preventive Medicine & Institute of Health Services Research, College of Medicine, Yonsei University, 250 Seongsan-no, Seodaemun-gu, Seoul, 120-752, Korea

## Abstract

**Background:**

Cancer screening rates in Korea for five cancer types have increased steadily since 2002. With regard to the life-time cancer screening rates in 2009 according to cancer sites, the second highest was breast cancer (78.1%) and the third highest was cervical cancer (76.1%). Despite overall increases in the screening rate, disparities in breast and cervical cancer screening, based on sociodemographic characteristics, still exist.

**Methods:**

Data from 4,139 women aged 40 to74 years from the 2005 to 2009 Korea National Cancer Screening Survey were used to analyze the relationship between sociodemographic characteristics and receiving mammograms and Pap smears. The main outcome measures were ever having had a mammogram and ever having had a Pap smear. Using these items of information, we classified women into those who had had both types of screening, only one screening type, and neither screening type. We used logistic regression to investigate relationships between screening history and sociodemographic characteristics of the women.

**Results:**

Being married, having a higher education, a rural residence, and private health insurance were significantly associated with higher rates of breast and cervical cancer screening after adjusting for age and sociodemographic factors. Household income was not significantly associated with mammograms or Pap smears after adjusting for age and sociodemographic factors.

**Conclusions:**

Disparities in breast and cervical cancer screening associated with low sociodemographic status persist in Korea.

## Background

Cancer screening rates in Korea for five cancer types have increased steadily since 2002 [1]. Regarding the life-time cancer screening rates in 2009, according to cancer site, the second highest was breast cancer (78.1%), and the third highest was cervical cancer (76.1%) [1]. A comparison of the cancer screening rates in Korea with those in other countries showed that the rates for breast (78.1%) and cervical cancer screening (76.1%) in Korea were lower than those in Great Britain (93 and 91%, respectively) [1,2].

In 1999, Korea began organized screening as part of the National Cancer Screening Program (NCSP), covering the entire population. NCSP invites women in Korea over the age of 40 years for breast cancer screening every 2 years, and women over the age of 30 years for cervical cancer screening every 2 years [1]. NCSP provides screening services free of charge for Medical Aid enrollees and people with National Health Insurance (NHI) with a contribution below 50%. Additionally, NCSP provides cancer screening to people with a contribution over 50% and has subsidized 90% of the costs of these services. The insurance contribution is calculated based on the individual's income level. In addition to the NCSP, cancer screening is conducted in outpatient clinics and private health assessment centers for opportunistic screening. However, individuals must pay for all procedure-related costs associated with such opportunistic screening [1].

Various studies have identified sociodemographic and health system-related characteristics that are barriers to or facilitators of breast and cervical cancer screening [3-10]. Well-established barriers to screening include sociodemographic characteristics, such as lower income, lower educational attainment, lack of appropriate health information, distance to services, fear of cancer, lack of health care insurance, and factors related to the healthcare system, such as lack of a recommendation for screening by a healthcare provider, poor coordination of services, poor access to transport, and lack of a patient or provider reminder system . Data from the USA indicated that the breast cancer screening rates of women in lower sociodemographic status were low, and that their morbidity and death rates have not been reduced [11-15]. A similar pattern emerges for cervical screening, with sociodemographic characteristics appearing to influence cervical screening rates in France and the United Kingdom (UK) as well as in urban areas of Australia [12,13,15-17].

Despite the overall increase in the screening rates, disparities in breast and cervical cancer screening based on sociodemographic characteristics still exist [3-10,18]. The objective of this study was to examine the relationships between sociodemographic characteristics and breast and cervical cancer screening among women in Korea.

## Methods

### Data sources

This study was performed using the Korean National Cancer Screening Survey (KNCSS) data from 2005 to 2009. KNCSS is a continuous national interview survey, conducted by the Korean National Cancer Center. KNCSS is conducted to investigate Korean participation rates in cancer screening for five common cancers: gastric, liver, colorectal, breast, and cervical cancer. Men and women were selected based on the Resident Registration Population data using a stratified, multistage, and random sampling procedure according to geographic area, age, and gender. The Resident Registration Population data are published annually by the Korea National Statistical Office after data are gathered from residents of the registration population every December 31. The publication provides data about changes in population size and structure and identifies population changes by administrative district. For the present study, investigators from a professional research agency conducted face-to-face interviews at the participants' homes. Study recruitment involved door-to-door contact. We made at least three attempts to contact a resident at each dwelling. Eligible participants were asked about their experiences of screening for five common cancers; health behaviors, health status, family history of cancer, and socioeconomic and demographic information. We included people from the age of 40 to 74 years in the KNCSS because those older than 75 years have difficulty recalling and answering many questions (*n *= 4,139). All subjects provided informed consent for participation in the study. This study was approved by the Institutional Review Board (IRB) of the National Cancer Center, Korea.

### Measures

For this study, variables of interest included age (40-49, 50-59, and 60-74), marital status (married or other (widowed, divorced, separated, or never married)), region of residence (metropolitan, urban, or rural), and private health insurance member (yes or no).

Education and household income were used to determine socioeconomic status. Education was classified into four categories: less than middle school (level 1), middle school graduate or some high school (level 2), high school graduate or some college (level 3), and college graduate or higher (level 4). Household income was categorized into four groups: < 1 million won per month (level 1), 1-2.5 (level 2), 2.5-4 (level 3), and > 4 (level 4) million won per month (1000 won ≈ US $0.84).

Those who did not attend were asked to choose one of eight reasons: had not heard about cancer screening, did not feel it was necessary, lacked time, could not afford cancer screening, feared the exam procedure, feared detecting cancer, had no faith in cancer screening, and no medical facilities in the neighborhood.

The main outcome measures were having ever had a mammogram and having ever had a Pap smear. Using these items of information, we classified women into those who had had both types of screening (that is, at least one mammogram and at least one Pap smear), only one screening type (a mammogram or a Pap smear, but not both), and neither screening type. We adopted this approach because women who have had one type of screening are known to be more likely to attend another screening program. We did not exclude women who had had a hysterectomy since the most recent Pap smear.

We analyzed data using the SAS software (ver. 9.1 for Windows). We calculated differences in breast and cervical cancer screening rates by age and sociodemographic factors. We used logistic regression to investigate relationships between screening history and sociodemographic characteristics of the women. We derived odds ratios (ORs) and 95% confidence intervals (CI) for categorical values. We regarded a *p*-value less than 0.05 as indicating statistical significance. We present both odds ratios adjusted for age only and fully adjusted odds ratios.

## Results

The response rates were 55.8-58.3% from 2005 to 2009 [18]. Of the participants, 55.8% reported having ever had a mammogram, and 75.5% reported having ever had a Pap smear (Table 1). Women with higher screening rates (having ever had a mammogram or Pap smear) were more likely to be age 50 or older, married, have a household income level of 4, and have private health insurance.

**Table 1:** Screening history by sociodemographic characteristics of respondents, 2005-2009

Variable	Number of women in sample	Ever had mammogram	Ever had Pap smear	Ever had both mammogram and Pap smear	Ever had either mammogram or Pap smear, not both	Never had either mammogram or Pap smear
	
	*n* (%)	*n* (%)	*n* (%)	*n* (%)	*n* (%)	*n* (%)
**Total**	**4139 (100.0)**	**2308 (55.8)**	**3123 (75.5)**	**2141 (51.7)**	**1148 (27.7)**	**849 (20.5)**
**Age**						
40-49	**1771 (100.0)**	**895 (50.5)**	**1373 (77.5)**	**848 (47.9)**	**572 (32.3)**	**351 (19.8)**
50-59	**1184 (100.0)**	**744 (62.8)**	**947 (80.0)**	**699 (59.0)**	**293 (24.8)**	**192 (16.2)**
60-74	**1184 (100.0)**	**669 (56.6)**	**803 (67.8)**	**594 (50.2)**	**283 (23.9)**	**306 (25.8)**
**Marital status**						
Married	**3621 (100.0)**	**2034 (56.2)**	**2795 (77.2)**	**1905 (52.6)**	**1018 (28.1)**	**697 (19.3)**
Other ^**a**^	**518 (100.0)**	**274 (52.9)**	**328 (65.3)**	**236 (45.6)**	**130 (25.1)**	**152 (29.3)**
**Residence**						
Metropolitan	**1930 (100.0)**	**1060 (54.9)**	**1462 (75.8)**	**990 (51.3)**	**542 (28.1)**	**398 (20.6)**
Urban	**1695 (100.0)**	**943 (55.6)**	**1271 (75.0)**	**870 (51.3)**	**474 (28.0)**	**351 (20.7)**
Rural	**514 (100.0)**	**305 (59.5)**	**390 (75.9)**	**281 (54.7)**	**132 (25.7)**	**100 (19.5)**
**Education **^**b**^						
Level 1	**1151 (100.0)**	**629 (54.7)**	**779 (67.7)**	**559 (48.6)**	**289 (25.1)**	**302 (26.2)**
Level 2	**786 (100.0)**	**466 (59.3)**	**596 (75.8)**	**430 (54.7)**	**202 (25.7)**	**154 (19.6)**
Level 3	**1796 (100.0)**	**977 (54.4)**	**1429 (79.6)**	**923 (51.4)**	**560 (31.2)**	**313 (17.4)**
Level 4	**406 (100.0)**	**236 (58.1)**	**319 (78.6)**	**229 (56.4)**	**97 (23.9)**	**80 (19.7)**
**Household income **^**c**^						
Level 1	**642 (100.0)**	**351 (54.7)**	**419 (65.3)**	**301 (46.9)**	**168 (26.2)**	**173 (27.0)**
Level 2	**1603 (100.0)**	**880 (54.9)**	**1206 (75.2)**	**823 (51.3)**	**439 (27.4)**	**340 (21.2)**
Level 3	**1292 (100.0)**	**711 (55.0)**	**1008 (78.0)**	**670 (51.9)**	**379 (29.3)**	**243 (18.8)**
Level 4	**602 (100.0)**	**366 (60.8)**	**490 (81.4)**	**347 (57.6)**	**162 (26.9)**	**93 (15.5)**
**Private health insurance member**						
Yes	**3011 (100.0)**	**1752 (58.2)**	**2401 (79.7)**	**1648 (54.7)**	**857 (28.5)**	**506 (16.8)**
No	**1128 (100.0)**	**556 (49.3)**	**722 (64.0)**	**493 (43.7)**	**291 (25.8)**	**343 (30.4)**

^a ^Other includes widowed, divorced, separated, and never married

^b ^Education: level 1, less than middle school; level 2, middle school graduate or some high school; level 3, high school graduate or some college; level 4, college graduate or higher

^c ^Household income: level 1, <1 million won per month; level 2, 1-2.5 million won per month; level 3, 2.5-4 million won per month; level 4, >4 million won per month (1000 won = US $0.84)

Table 2 shows the odds ratio of receiving a mammogram or Pap smear, adjusted for age and sociodemographic factors (marital status, region of residence, education, household income, and private health insurance). Positive associations were found between education and both mammogram and Pap smear screenings. For example, those with an education level of 4 were more likely to have had screening procedures than were those with an education level of 1; after adjustment for age, the odds ratios were 1.62 (95% CI = 1.24-2.13) for mammograms and 1.60 (95% CI = 1.17-2.20) for Pap smears. Those with a household income level of 4 were more likely to have had screening procedures than were those with a household income level of 1; after adjustment for age, the odds ratios were 1.54 (95% CI = 1.20-1.97) for mammograms and 1.91 (95% CI = 1.43-2.54) for Pap smears. After adjusting for age and sociodemographic factors, private health insurance was the only significant predictor when we compared women who had had mammograms with those who had not (*p *< 0.0001). Marital status (*p *< 0.0001) and private health insurance (*p *< 0.0001) were significant predictors of having had a Pap smear versus having had none.

**Table 2:** Odds ratios of screening history (ever had a mammogram, ever had a Pap smear) by sociodemographic characteristics, 2005-2009

Variable	Odds ratio (95% CI)			
	
	Ever versus never had mammogram		Ever versus never had Pap smear	
	
	Age adjusted	Fully adjusted*	Age adjusted	Fully adjusted*
**Marital status**				
Married	**1.00**	**1.00**	**1.00**	**1.00**
Other ^**a**^	**0.81 (0.66-0.98)**	**0.87 (0.69-1.11)**	**0.62 (0.50-0.76)**	**0.64 (0.50-0.82)**
*p*-value		**0.151**		**< .0001**
**Residence**				
Metropolitan	**1.00**	**1.00**	**1.00**	**1.00**
Urban	**1.04 (0.91-1.18)**	**1.04 (0.90-1.21)**	**0.99 (0.85-1.16)**	**0.97 (0.82-1.16)**
Rural	**1.18 (0.97-1.46)**	**1.29 (1.02-1.62)**	**1.10 (0.87-1.38)**	**1.17 (0.89-1.52)**
*p*-value		**0.059**		**0.547**
**Education **^**b**^				
Level 1	**1.00**	**1.00**	**1.00**	**1.00**
Level 2	**1.31 (1.07-1.59)**	**1.27 (1.01-1.60)**	**1.33 (1.07-1.66)**	**1.22 (0.94-1.58)**
Level 3	**1.30 (1.07-1.58)**	**1.15 (0.90-1.46)**	**1.67 (1.33-2.09)**	**1.36 (1.03-1.79)**
Level 4	**1.62 (1.24-2.13)**	**1.35 (0.98-1.87)**	**1.60 (1.17-2.20)**	**1.17 (0.80-1.70)**
*p*-value		**0.213**		**0.182**
**Household income **^**c**^				
Level 1	**1.00**	**1.00**	**1.00**	**1.00**
Level 2	**1.10 (0.90-1.33)**	**0.98 (0.78-1.24)**	**1.39 (1.12-1.71)**	**1.15 (0.89-1.48)**
Level 3	**1.16 (0.94-1.44)**	**0.97 (0.74-1.25)**	**1.53 (1.21-1.93)**	**1.13 (0.85-1.51)**
Level 4	**1.54 (1.20-1.97)**	**1.18 (0.87-1.60)**	**1.91 (1.43-2.54)**	**1.36 (0.96-1.93)**
*p*-value		**0.297**		**0.163**
**Private health insurance member**				
Yes	**1.00**	**1.00**	**1.00**	**1.00**
No	**0.58 (0.49-0.69)**	**0.60 (0.50-0.73)**	**0.48 (0.40-0.58)**	**0.54 (0.44-0.66)**
*p*-value		**< .0001**		**< .0001**

^a ^Other includes widowed, divorced, separated, and never married

^b ^Education: level 1, less than middle school; level 2, middle school graduate or some high school; level 3, high school graduate or some college; level 4, college graduate or higher

^c ^Household income: level 1, <1 million won per month; level 2, 1-2.5 million won per month; level 3, 2.5-4 million won per month; level 4, >4 million won per month (1000 won = US $0.84)

*Adjusted for age, marital status, education, household income, residence, and private health insurance

Being married, having a rural residence, having an education level of 4, and being a private health insurance member were significant predictors of having had both a mammogram and Pap smear, compared with having had only one or neither of these screenings, after adjustment for age and sociodemographic factors. In particular, an education level of 4 had an odds ratio of 1.51 (95% CI = 1.10-2.08) compared with an education level of 1 in ever having had both a mammogram and Pap smear versus having had only one or neither screening, after adjustment for age and sociodemographic factors. Marital status (*p *= 0.003) and private health insurance (*p *< 0.0001) were significant predictors of having had some screening compared with having had none, after adjustment for age and sociodemographic factors. These results enable us to investigate who was likely to participate in screening (Table 3).

**Table 3:** Odds ratios of screening history (ever had both screenings, ever had some screening) by sociodemographic characteristics, 2005-2009

Variable			Odds ratio (95% CI)			
			
	No. in sample: ever both/ever some (*n *= 2141/3290)		Ever had both mammogram and Pap smear versus not both*		**Ever had mammogram, Pap smear, or both versus never had either**†	
			
			Age adjusted	**Fully adjusted**‡	Age adjusted	**Fully adjusted**‡
**Marital status**						
Married	**1905**	**/2924**	**1.00**	**1.00**	**1.00**	**1.00**
Other ^**a**^	**236**	**/366**	**0.73 (0.60-0.89)**	**0.78 (0.62-0.99)**	**0.66 (0.53-0.83)**	**0.70 (0.54-0.91)**
*p*-value				**0.012**		**0.003**
**Residence**						
Metropolitan	**990**	**/1532**	**1.00**	**1.00**	**1.00**	**1.00**
Urban	**870**	**/1344**	**1.01 (0.89-1.16)**	**1.02 (0.88-1.18)**	**1.02 (0.87-1.20)**	**1.00 (0.83-1.20)**
Rural	**281**	**/414**	**1.15 (0.94-1.40)**	**1.30 (1.03-1.63)**	**1.14 (0.89-1.45)**	**1.15 (0.87-1.53)**
*p*-value				**0.071**		**0.546**
**Education **^**b**^						
Level 1	**559**	**/849**	**1.00**	**1.00**	**1.00**	**1.00**
Level 2	**430**	**/632**	**1.33 (1.09-1.62)**	**1.26 (1.00-1.59)**	**1.35 (1.07-1.72)**	**1.27 (0.96-1.69)**
Level 3	**923**	**/1483**	**1.38 (1.13-1.67)**	**1.19 (0.94-1.51)**	**1.64 (1.29-2.08)**	**1.34 (1.00-1.80)**
Level 4	**229**	**/326**	**1.80 (1.38-2.36)**	**1.51 (1.10-2.08)**	**1.45 (1.05-2.02)**	**1.01 (0.68-1.49)**
*p*-value				**0.055**		**0.551**
**Household income **^**c**^						
Level 1	**301**	**/469**	**1.00**	**1.00**	**1.00**	**1.00**
Level 2	**823**	**/1263**	**1.24 (1.02-1.51)**	**1.09 (0.87-1.38)**	**1.21 (0.97-1.52)**	**1.00 (0.77-1.31)**
Level 3	**670**	**/1049**	**1.32 (1.07-1.63)**	**1.05 (0.81-1.36)**	**1.35 (1.05-1.74)**	**1.02 (0.75-1.39)**
Level 4	**347**	**/509**	**1.73 (1.36-2.22)**	**1.24 (0.91-1.68)**	**1.75 (1.29-2.38)**	**1.34 (0.92-1.96)**
*p*-value				**0.263**		**0.180**
**Private health insurance member**						
Yes	**1648**	**/2505**	**1.00**	**1.00**	**1.00**	**1.00**
No	**493**	**/785**	**0.06 (0.47-0.67)**	**0.60 (0.50-0.72)**	**0.46 (0.37-0.56)**	**0.50 (0.41-0.62)**
*p*-value				**< .0001**		**< .0001**

^a ^Other includes widowed, divorced, separated, and never married

^b ^Education: level 1, less than middle school; level 2, middle school graduate or some high school; level 3, high school graduate or some college; level 4, college graduate or higher

^c ^Household income: level 1, <1 million won per month; level 2, 1-2.5 million won per month; level 3, 2.5-4 million won per month; level 4, >4 million won per month (1000 won = US $0.84)

* Compares women who have had both screening types with those who have had only one screening type or neither screening (that is, both screening versus not both)

† Compares women who have had either or both screening types with those who have had neither screening (that is, some screening versus none)

‡ Adjusted for age, marital status, education, household income, residence, and private health insurance

**Figure 1:**
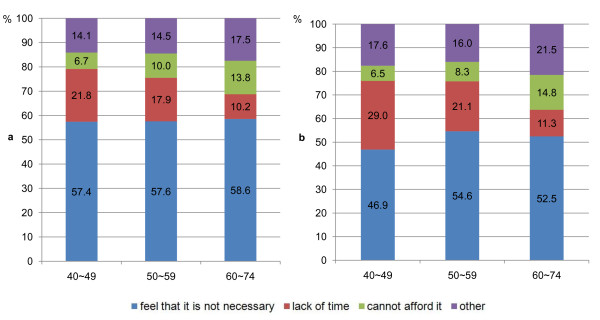
**Reasons for not having had a mammogram (a), and a Pap smear (b), women aged 40-74 years**.

The three most common reasons women gave for not having had a mammogram or Pap smear were, in all age groups: 1) they did not feel it was necessary, 2) they did not have enough time, and 3) they could not afford it (Figure 1).

## Discussion

The findings of this study contribute to our understanding of the sociodemographic characteristics associated with the use of breast and cervical cancer screening. Being married and having a higher education level, rural residence, and private health insurance were significantly associated with higher rates of breast and cervical cancer screening, after adjusting for age and sociodemographic factors. Household income was not significantly associated with mammograms or Pap smears.

Of the sociodemographic factors, household income was not shown to be significantly associated with mammograms or Pap smears by multivariate logistic regression after full adjustment. Other studies have suggested that household income affects mammogram and Pap smear participation, with women from low-income households less likely to participate than those from high-income households [2,5,19-21]. Inequalities in breast and cervical cancer screening still exist in the UK, despite free screening for the entire population [2]. Many studies have suggested that having access to a physician who performs mammograms and Pap smears was a powerful predictor of breast and cervical cancer screening [2,5,21-26]. A possible cause of this difference in study results is that in 1999, Korea began screening for cancer as part of the NCSP, which covers the entire population. NCSP provides screening services free of charge for Medical Aid enrollees and NHI participants with a contribution below 50%. Since 2010, the NCSP has included a subsidy of 90% for people with NHI with a contribution over 50%. Such government support might have reduced the effect of household income on breast and cervical cancer screening participation [1,18,27].

Our finding of higher rates of having ever had a mammogram and Pap smear among women with a rural residence differs from the results of other studies that have indicated low rates among women with a rural residence [5,23-25]. This may have resulted partly from the mobile screening service now provided by the NCSP. The mobile screening service is helpful for target populations who are not able to access medical institutions to obtain appropriate screening, and it may contribute to improving compliance with the screening program. The increase in the compliance rate for the cancer screening program might have resulted from the provision of accessible and acceptable screening services, such as mobile screening.

A disparity in mammogram and Pap smear use was found among women of different education levels after adjusting for age and sociodemographic factors. Other studies have used multivariate logistic regression analysis to show that women were more likely to undergo a mammogram and Pap smear if they had a higher education level [2,5,19-21]. To date, a low education level is a known barrier to breast and cervical cancer screening. Some studies have indicated that routine monitoring of coverage of screening and information polices affect breast and cervical cancer screening rates at various education levels [2,18,22,24]. Additionally, the perception of not needing the test due to good health or an absence of symptoms was the most frequently reported barrier to participation in breast and cervical cancer screening in all age groups. Thus, we need to increase the knowledge and awareness of cancer in the target population to increase the participation rate in cancer screening programs [22,24,26,28-30]. Attempts to promote cancer screening have used a public health model that targets entire communities, *e.g*., mass-media campaigns about the organized screening system in Korea. Additional individual-directed interventions in health care settings regarding cancer screening use are required, such as individualized in-person or telephone counseling, individualized letters and reminders, or other individual-directed strategies, to increase participation and reduce the disparity in cancer screening [18,27,30].

There may be other reasons for the low perceived risk of breast and cervical cancer in addition to perceptions of good health or an absence of symptoms. There could be no experience of cancer among friends and family, misperceptions about the causes of cancer, or not feeling at risk of cervical cancer because of sexual experience [22,24,25]. Alternative reasons could include the fact that the service offered is unattractive to women or promoted in an unattractive manner. However, we did not investigate these reasons in this study. We need to study these reasons further. The rate of not undergoing screening of breast and cervical cancer due to a lack of time was high in the women between 40 and 49 years old compared with other age groups. Officials are discussing whether to give a holiday for cancer screening or to provide cancer screening service at the employee's place of work while on duty.

Private health insurance was the strongest predictor of breast and cervical cancer screening. Koreans can take cancer screening through organized or opportunistic systems. Even if they can take cancer screening free of charge or for a small fee, which is only 10% of the cost, when they want to take organized cancer screening, some people prefer opportunistic screening to organized screening. In this case, having private health insurance is a necessary precondition for improving the use of cancer screening, because private health insurance can remove economic and practical barriers to screening in opportunistic settings [31].

This study has several limitations, based on the KNCSS data that we used. First, KNCSS data were self-reported, which may have introduced a bias because several studies have suggested that self-reports overestimate the prevalence of participation in cancer screening. Second, we were unable to explore the influence of other important correlates, such as test-specific characteristics (*e.g*., preparation, cost, time constraints, and transportation for screening) and psychological factors (*e.g*., discomfort, concern about complications, or anxiety about the procedure) involved in the use of breast and cervical cancer screening. Third, we focused on women who have ever had screening in this study. It is difficult to compare the life-time screening rates with screening rates with recommendations directly.

## Conclusions

In summary, we found that married marital status, higher educational level, rural residence, and private health insurance were significantly associated with higher rates of breast and cervical cancer screening after adjustment for age and sociodemographic factors. To improve the participation rate for breast and cervical cancer screening, more attention should be given to women in lower sociodemographic groups. Future analyses of the use of breast and cervical cancer screening for women could include the influence of other important correlates, such as test-specific characteristics (*e.g*., preparation, cost, time constraints, or transportation for screening) and psychological factors (*e.g*., discomfort, concern about complications, or anxiety about the procedure) in greater detail in the Korean National Cancer Screening Survey data.

## Competing interests

The authors declare that they have no competing interests.

## Authors' contributions

MP participated in the design of the study and drafted the manuscript. EP participated in the design of the study. KC participated in the sequence alignment. JJ performed the statistical analyses. HL participated in the design of the study and drafted the manuscript. All authors read and approved the final manuscript.

## Pre-publication history

The pre-publication history for this paper can be accessed here:

http://www.biomedcentral.com/1471-2407/11/257/prepub
